# Paternal chronic colitis causes epigenetic inheritance of susceptibility to colitis

**DOI:** 10.1038/srep31640

**Published:** 2016-08-19

**Authors:** Markus Tschurtschenthaler, Priyadarshini Kachroo, Femke-Anouska Heinsen, Timon Erik Adolph, Malte Christoph Rühlemann, Johanna Klughammer, Felix Albert Offner, Ole Ammerpohl, Felix Krueger, Sébastien Smallwood, Silke Szymczak, Arthur Kaser, Andre Franke

**Affiliations:** 1 Division of Gastroenterology and Hepatology, Department of Medicine, University of Cambridge, CB2 0QQ Cambridge, United Kingdom; 2 Department of Medicine II (Gastroenterology & Hepatology), Medical University Innsbruck, 6020 Innsbruck, Austria; 3 Institute of Clinical Molecular Biology, Christian-Albrechts-University Kiel, 24105 Kiel, Germany; 4 CeMM Research Center for Molecular Medicine of the Austrian Academy of Sciences, 1090 Vienna, Austria; 5 Department of Pathology, Academic Teaching Hospital Feldkirch, 6800 Feldkirch, Austria; 6 Institute of Human Genetics, Christian-Albrechts-University Kiel, 24105 Kiel, Germany; 7 Bioinformatics Group, Babraham Institute, CB22 3AT Cambridge, United Kingdom; 8 Epigenetics Programme, Babraham Institute, CB22 3AT Cambridge, United Kingdom

## Abstract

Inflammatory bowel disease (IBD) arises by unknown environmental triggers in genetically susceptible individuals. Epigenetic regulation of gene expression may integrate internal and external influences and may thereby modulate disease susceptibility. Epigenetic modification may also affect the germ-line and in certain contexts can be inherited to offspring. This study investigates epigenetic alterations consequent to experimental murine colitis induced by dextran sodium sulphate (DSS), and their paternal transmission to offspring. Genome-wide methylome- and transcriptome-profiling of intestinal epithelial cells (IECs) and sperm cells of males of the F_0_ generation, which received either DSS and consequently developed colitis (F_0_^DSS^), or non-supplemented tap water (F_0_^Ctrl^) and hence remained healthy, and of their F_1_ offspring was performed using reduced representation bisulfite sequencing (RRBS) and RNA-sequencing (RNA-Seq), respectively. Offspring of F_0_^DSS^ males exhibited aberrant methylation and expression patterns of multiple genes, including *Igf1r* and *Nr4a2*, which are involved in energy metabolism. Importantly, DSS colitis in F_0_^DSS^ mice was associated with decreased body weight at baseline of their F_1_ offspring, and these F_1_ mice exhibited increased susceptibility to DSS-induced colitis compared to offspring from F_0_^Ctrl^ males. This study hence demonstrates epigenetic transmissibility of metabolic and inflammatory traits resulting from experimental colitis.

The inflammatory bowel diseases (IBD) Crohn’s disease (CD) and ulcerative colitis (UC) are triggered by yet unknown environmental factors in genetically susceptible individuals^1^,^2^,^3^. IBD has dramatically risen in incidence over the last decades in the Western world, and more recently in countries adopting Western lifestyles^1^. Concordance rates in monozygotic twins of 40–50% and 10–15% in CD and UC^4^,^5^,^6^,^7^, respectively, indicate the relative contribution of genetic *vs.* environmental risks. In the last years genome-wide association studies (GWAS) identified thousands of single-nucleotide polymorphisms (SNPs) associated with complex diseases, and have been particularly successful in CD and UC^8^. It is estimated that only approximately 16% of IBD disease heritability can be explained by known common genetic risk factors^9^,^10^. While genetic interactions, which are not accounted for in these estimates, but which are consistent with observable data in human complex disease genetics^11^ and in model systems^12^, may create ‘phantom heritability’ and hence may possibly explain a large part of the current ‘missing heritability’^13^, additional, non-genetic, environmentally-imprinted disease triggers, e.g. epigenetic modifications, could contribute to the heritable fraction of disease risk.

Epigenetic alterations, including methylation, histone modifications and RNA-mediated effects, regulate gene expression, whereas the DNA sequence itself remains unaltered. DNA becomes predominantly methylated at cytosines (CpG sites or CpGs), but methylation at non-CpG sites has also been detected^14^,^15^,^16^. The highly dynamic nature, cell-specific patterns and genomic context of DNA methylation are of great importance in cellular and metabolic processes, development and disease^17^,^18^. Recent studies showed aberrant DNA methylation profiles in intestinal tissues of IBD patients^19^,^20^,^21^,^22^,^23^,^24^,^25^. Importantly, some epigenetic marks are acquired during periods of developmental plasticity and are not completely erased during epigenetic reprogramming of non-imprinted genes after fertilization allowing them to being passed on to the next generation^26^,^27^,^28^,^29^. Using this mechanism of inheritance, epigenomes of gametes serve as ‘messengers of ancestral exposures’ (epigenetic memory) and ‘inform’ offspring about prevailing environmental conditions^30^,^31^,^32^,^33^. Some of the recent studies report a maternal inheritance of epigenetic effects, which, however, are difficult to separate from *in utero* exposure or from other factors that can be passed on to the next generations via social or cultural inheritance systems^30^. In contrast, studying epigenetic inheritance in the paternal system has the advantage that sperm material likely serves as the only ‘carrier’ of epigenetic information to the next generation. However, in mammals, paternal and maternal methylomes go through active de-methylation at the majority of CpGs^34^. Only a few rare methylation sites can escape this process of ‘epigenetic reprogramming’ and can be passed on to the next generation^35^. Interestingly, a human cohort study investigating paternal epigenetic inheritance demonstrated that parental grandfathers, who were overfed in their childhood, had grandchildren with an increased mortality, a higher risk of diabetes and cardiovascular diseases^36^. Furthermore, a recent study investigating the role of DNA methylation in a trans-generational mouse model of undernourishment showed that under-nutrition during prenatal life perturbed epigenetic reprogramming of sperms with concomitant DNA methylation changes in the adult brain and liver tissues, which in turn were associated with metabolic disease (early-life adiposity, impaired pancreatic function, glucose intolerance) in offspring^37^. Moreover, a recent study in mice suggested that chronic paternal high fat diet (HFD) reprograms pancreatic β-cell function in their female offspring, which leads to impaired glucose-insulin homeostasis^28^. Similarly, a paternal low-protein diet can affect offspring metabolism by increasing expression of many hepatic genes involved in lipid and cholesterol biosynthesis, which correlated with aberrant cytosine methylation in offspring of males fed with a low-protein diet^30^. In addition, paternally inherited effects were linked to obesity^38^,^39^,^40^,^41^, diabetes^29^,^42^, glucose metabolism^43^, hepatic wound healing^44^, behavioral and stress responses^45^,^46^,^47^,^48^,^49^,^50^ and toxin-inherited effects^51^,^52^,^53^. These studies suggest that the paternal lifestyle permanently influenced by certain environmental factors can affect spermatogenesis and can induce inter-generational transmission of epigenetic marks modulating offspring’s metabolism, contributing to disease risk.

To the best of our knowledge, there is no study to date that has investigated an inter-generational transmission of epigenetic marks in the context of chronic intestinal inflammation. We established a colitis mouse model, which allowed us to elucidate the impact of a chronic environmentally-induced colitis on intestinal DNA methylation and the proposed transmission of aberrant methylation patterns to offspring.

## Results

### Offspring of DSS colitic males display a lower body weight and increased susceptibility to colitis

**Figure 1:**
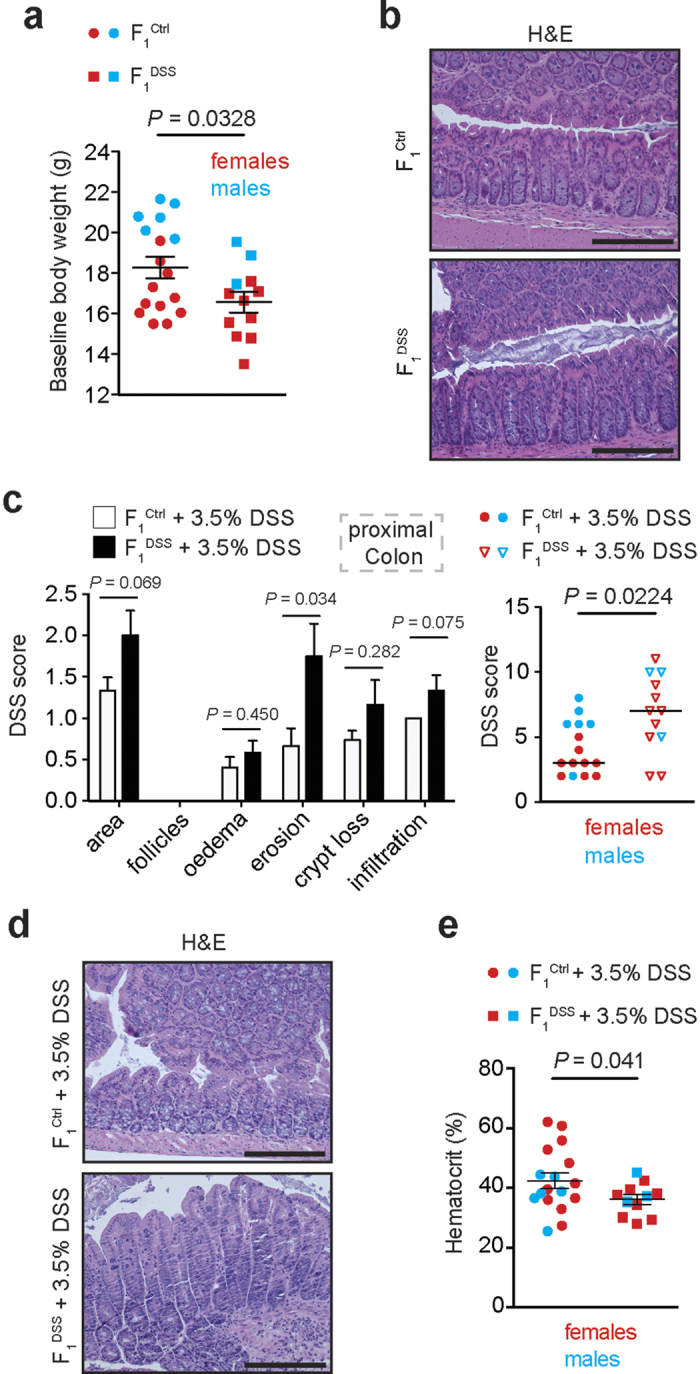
Chronic experimental colitis leads to a lower body weight and increased susceptibility to inflammation in the offspring. (**a**) Body weight comparison between 7 week-old female and male littermates that are offspring of either F_0_^Ctrl^ males (F_1_^Ctrl^) or F_0_^DSS^ males (F_1_^DSS^). Mice littermates of both groups were taken from average litter sizes: F_1_^Ctrl^ and F_1_^DSS^ mice originated from 3 and 2 different litters, respectively. F_1_^Ctrl^ females, n = 11; F_1_^DSS^ females, n = 9; F_1_^Ctrl^ males, n = 6; F_1_^DSS^ males, n = 3. Mean ± SEM; Unpaired, two-tailed Student’s t-test. (**b**) Representative H&E stainings of the distal colon of 13 week-old F_1_^Ctrl^ and F_1_^DSS^ mice at baseline conditions (scale bars, 200 μm). (**c**) DSS subscores (left) and the resulting cumulative score (right) of the proximal colon of F_1_^Ctrl^ and F_1_^DSS^ mice after induction of an acute colitis by adding 3.5% DSS in the drinking water of mice of both groups (F_1_ DSS susceptibility test). F_1_^Ctrl^ females, n = 9; F_1_^DSS^ females, n = 9; F_1_^Ctrl^ males, n = 6; F_1_^DSS^ males, n = 3. Mean ± SEM; Mann–Whitney *U*-test. (**d**) Representative H&E stainings of the proximal colon of F_1_^Ctrl^ and F_1_^DSS^ females after 6 days of treatment with 3.5% DSS (scale bars, 200μm). (**e**) Blood hematocrit value (in %) of F_1_^Ctrl^ and F_1_^DSS^ mice after 3.5% DSS treatment. F_1_^Ctrl^ females, n = 11; F_1_^DSS^ females, n = 8; F_1_^Ctrl^ males, n = 6; F_1_^DSS^ males, n = 3; Mean ± SEM; Unpaired, 1-tailed Student’s t-test.

To decipher epigenetically altered marks that might contribute to the etiopathogenesis of colitis, we exposed wildtype C57Bl/6(N) male littermate mice at 5 weeks of age (Supplementary Fig. 1a) to three cycles of dextran sodium sulphate (DSS), which resulted in body weight loss and the induction of a chronic colitis in F_0_^DSS^ mice compared to their F_0_^Ctrl^ littermates that were meanwhile kept on tap water (Supplementary Fig. 1b). After recovering from the last DSS cycle, males of both groups were mated overnight with healthy female littermates (Supplementary Fig. 1a), before the colon was harvested for histology and length assessment, which is a measure for the extent of inflammation (Supplementary Fig. 1c–e). Next, we compared the offspring of DSS-treated males (‘F_1_^DSS^’) with the offspring of healthy males not exposed to DSS (F_1_^Ctrl^) (Supplementary Fig. 1f). The litter sizes between offspring of F_0_^Ctrl^ and F_0_^DSS^ mice were comparable (data not shown). However, the body weight of male and female offspring of F_0_^DSS^ mice (‘F_1_^DSS^’) was significantly lower compared to offspring of F_0_^Ctrl^ mice (‘F_1_^Ctrl^’; Fig. 1a). F_1_^DSS^ mice at the age of 14 weeks exhibited a morphologically healthy colon (Fig. 1b). We next studied whether chronic intestinal inflammation in the paternal generation affects their offspring’s susceptibility to acute DSS colitis. For this purpose we applied 3.5% DSS to the drinking water of 7 week-old F_1_^DSS^ and F_1_^Ctrl^ mice for 6 consecutive days. Notably, F_1_^DSS^ mice exhibited higher colitis scores in the proximal colon (Fig. 1c,d) and lower hematocrit levels (Fig. 1e) compared to offspring of healthy F_0_^Ctrl^ mice. Interestingly, female offspring exhibited more pronounced effects compared to male offspring (Supplementary Fig. 2a–d). We noted no differences in the severity of inflammation in the distal colon of F_1_^DSS^ compared to F_1_^Ctrl^ mice, which, however, was severely inflamed which may have obfuscated our ability to discern modest differences (Supplementary Fig. 2e,f). Weight loss during DSS colitis and colonic length were comparable between F_1_^DSS^ and F_1_^Ctrl^ mice (Supplementary Fig. 2g,h). In summary, chronic DSS colitis in the F_0_ generation was associated with lower body weight and modestly increased susceptibility to acute DSS colitis in their F_1_ offspring, when compared to offspring from non-colitic mice.

### Chronic inflammation induced by DSS leads to a differential DNA methylation pattern in sperm cells of the F_0_ and F_1_ generation

The reduced body weight and increased DSS-susceptibility of F_1_^DSS^ mice suggested the involvement of epigenetic inheritance from F_0_^DSS^ males to their offspring. In order to test this, we analyzed sperm cells together with fluorescence-activated cell sorted (FACS) EpCAM^+^ CD45^−^ colonic intestinal epithelial cells (IECs), which are primarily affected in DSS-induced colitis^54^, from mice of the F_1_ and F_0_ generation (Supplementary Fig. 1f and Supplementary Fig. 3a) and subjected them to RNA-sequencing (RNA-Seq) and reduced representation bisulfite sequencing (RRBS) to assess their global mRNA expression and methylation profile, respectively. The normalized gene expression counts (Supplementary Fig. 3b) of epithelial (*Actb*, *Cdh1/EpCAM*) and sperm markers (*Odf1*, *Smcp*) confirmed the purity of samples used in this study (Supplementary Fig. 3c–h). Moreover, multi-dimensional scaling (MDS) analysis of the filtered and preprocessed methylome data showed clear differences between epithelial and sperm cells (Supplementary Fig. 4a).

**Figure 2:**
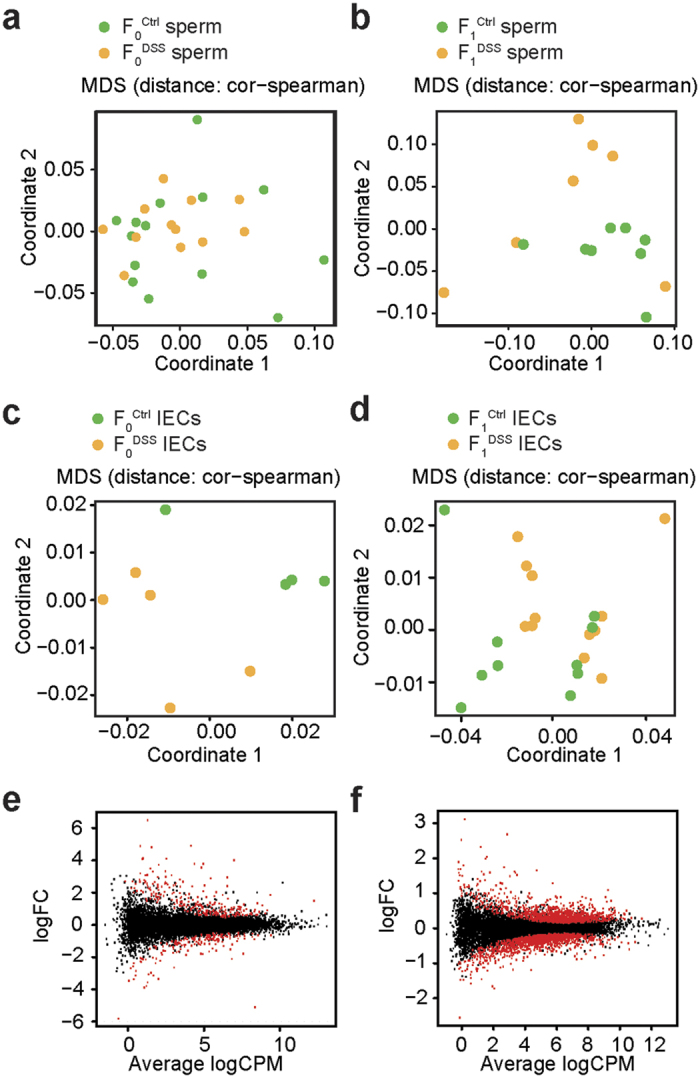
DSS treatment affects gene expression in the F_1_ generation. (**a**) MDS plot showing global effect of DSS treatment on methylation in sperms of F_0_^Ctrl^ and F_0_^DSS^ mice. (**b**) MDS plot showing global effect of DSS treatment in the F_0_ generation on methylation in sperms of F_1_^Ctrl^ and F_1_^DSS^ mice. (**c**) MDS plot showing global effect of DSS treatment on gene expression in colonic IECs of the F_0_ generation. (**d**) MDS plot showing global effect of DSS treatment in the F_0_ generation on gene expression of IECs in the F_1_ generation based on filtered high quality gene expression counts (*P* = 0.034, multivariate ANOVA). (**e**) MA plot showing the effect size and the significance (red colored dots) for F_0_^Ctrl^ and F_0_^DSS^ IECs. Differentially expressed genes (up-regulated or down-regulated) are highlighted in red color. (**f** ) MA plot showing the effect size and the significance for F_1_^Ctrl^ and F_1_^DSS^ IECs. Differentially expressed genes (up-regulated or down-regulated) are highlighted in red color.

Importantly, MDS analysis of the quality-controlled and filtered CpG sites in F_0_ and F_1_ sperm samples (F_0_: 820,122 CpG sites *vs.* F_1_: 727,273 CpG sites identified) pointed to stronger methylation differences in the F_1_ generation compared to the F_0_ generation (Fig. 2a,b). Comparing sperm samples of F_0_^Ctrl^ and F_0_^DSS^ mice resulted in 823 differentially methylated CpG sites with a methylation difference >0.20 (477 hypo-methylated and 346 hyper-methylated). Notably, 410 of these CpG sites could be annotated to either transcripts and/or promoters (Supplementary Data 1). Surprisingly, comparing the DNA methylation pattern of sperm cells of F_1_^Ctrl^ mice with sperm cells of F_1_^DSS^ mice resulted in 4,617 differentially methylated sites (2,429 hypo-methylated and 2,188 hyper-methylated) of which 1,680 CpG sites could be assigned to a specific gene (Supplementary Data 2). The quality controlled filtered CpGs in both generations were annotated to mainly regions outside of CpG islands (CGIs), inter-genic regions and regulatory regions of CTCF-binding sites (transcriptional repressor 11-zinc finger protein or CCCTC-binding factor) (Supplementary Fig. 4b,c).

We identified an overlap of 66 significantly differentially methylated genes (Supplementary Data 3) between sperm samples of mice of the F_0_ and F_1_ generation (37 hypo-methylated and 29 hyper-methylated genes). Importantly, this number is significantly larger than expected by chance in all 10,000 permutations (mean number after 10,000 permutations = 15.518 [3, 30]; Permutation based p-value = 0) (Supplementary Fig. 5a). The 50 most differentially methylated genes overlapping between sperm samples of both generations are shown as heatmaps in Supplementary Fig. 5b,c. Analyzing all 66 overlapping genes, ten CpG sites showed a differential methylation within the promoter or the transcription factor binding region of their annotated genes (*Gm128, Pnpla1, Plekhg4, Hdac5, Tjp3, Ttc28, Pnpla1, Mir6991, Hmha1, Gm6484*) and two CpGs were annotated to regulatory enhancers near *Igf1r* and *Mcf2l*. Moreover, seven differentially methylated sites were located at the same genomic location in both generations, two of which, were present within the intron or exon of the genes *Mta1* and *Zfp865*, respectively. The remaining five sites (chr10:12,2960,510, chr15:37,868,157, chr17:7,882,215, chr4:136,419,115, chr19:16,938,303) were outside of CGIs (Supplementary Data 1 and Supplementary Data 2). Collectively, these data suggest that DSS-induced colitis in male mice affects the DNA methylation pattern in their and their offspring’s sperm cells.

### Offspring of DSS-treated males display differential gene expression in colonic IECs at baseline conditions pointing to a dysregulation of the immune response

**Figure 3:**
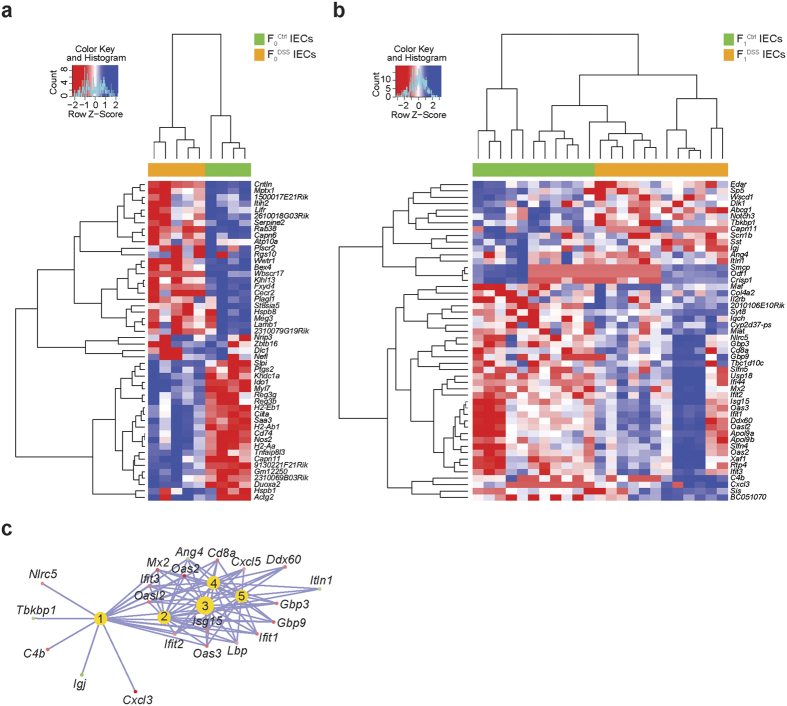
Differential gene expression in IECs of the F_1_ generation induced by chronic inflammation in the F_0_ generation. (**a**) Heatmap showing hierarchical clustering of the top 50 differentially expressed genes (according to fold changes) in IECs between F_0_^Ctrl^ and F_0_^DSS^ mice. (**b**) Heatmap showing hierarchical clustering of the top 50 differentially expressed genes in IECs (according to fold changes) between F_1_^Ctrl^ and F_1_^DSS^ mice. Red and blue boxes indicate down-regulated and up-regulated genes, respectively. (**c**) Gene enrichment analysis of differentially expressed genes in the F_1_ generation (*P*_adjusted _< 0.05, absolute FC > 1). 1 = immune response, 2 = response to external biotic stimulus, 3 = defense response to other organism, 4 = response to other organism, 5 = response to biotic stimulus.

To explain if the differences in the sperm methylome contribute to a potential gene dysregulation in the F_1_^DSS^ epithelium, which could possibly contribute to reduced body weight and increased susceptibility to colitis (Fig. 1a,c–e), we assessed the global mRNA expression profile of EpCAM^+^ CD45^−^ colonic IECs of mice of the F_0_ and F_1_ generation with the latter not being exposed to DSS. MDS analysis of the mRNA expression profile not only showed a significant separation of F_0_^DSS^ and F_0_^Ctrl^ IECs (Fig. 2c) as expected, but notably also of F_1_^DSS^ and F_1_^Ctrl^ IECs (Fig. 2d). Comparative gene expression analysis of IECs of F_0_^DSS^ and F_0_^Ctrl^ IECs revealed 230 statistically significantly differentially expressed genes (*P*_adjusted _< 0.05; 13,198 tested genes with mean coverage of 30.83x) (Supplementary Data 4, Fig. 2e), whereas 2,358 differentially expressed genes were detected between F_1_^DSS^ and F_1_^Ctrl^ IECs (*P*_adjusted _< 0.05; Supplementary Data 5, Fig. 2f). The 50 most differentially expressed genes (based on their fold changes) of IECs of the F_0_ generation and F_1_ generation are shown as heatmaps in Fig. 3a,b, respectively. Gene set enrichment analysis for the differentially expressed genes in the F_1_ generation points to a strong deregulation of the immune response in F_1_^DSS^ epithelium involving key genes of the type I IFN response (*Tbkbp1, Gbp3, Gbp9, Ifit1, Ifit2, Ifit3, Oas2, Oas3, Isg15, Oasl2, Ddx60, Mx2*). This analysis also identified key genes involved in immune pathways and pro-inflammatory processes (*Cxcl3, Cxcl5, Cd8a, C4b, Igj, Nlrc5, Lbp*), energy metabolism (*Itln1*) and vascular development (*Ang4*) (Fig. 3c). Taken together, offspring of male mice that experienced chronic colitis exhibited an altered intestinal epithelial transcriptome, which highlights a dysregulation of immunological and inflammatory pathways.

### Differential DNA methylation in colonic epithelial cells

**Figure 4:**
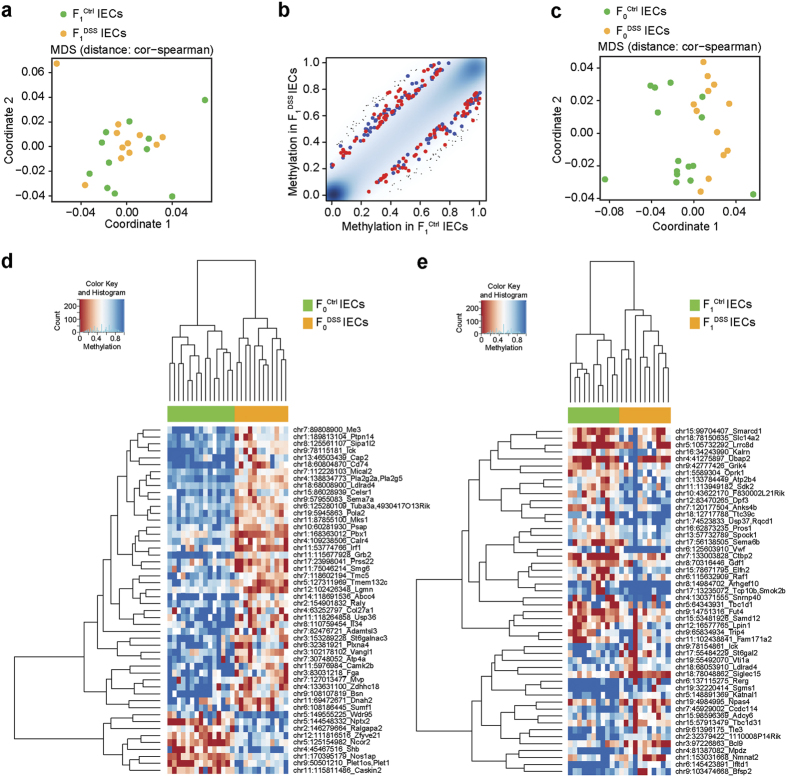
DSS-induced methylation changes are transmissible to offspring leading to a differential methylation profile in colonic epithelial cells. (**a**) MDS showing global effect of DSS treatment on methylation in IECs of F_1_^Ctrl^ and F_1_^DSS^ mice. (**b**) Global methylation in F_1_ IECs is shown for methylated CpG sites in F_1_^Ctrl^ (x-axis) compared to F_1_^DSS^ mice (y-axis). Blue and red dots indicate significant CpG sites associated with *P *< 0.05 and a methylation difference >0.20. (**c**) MDS plot showing global effect of DSS treatment on methylation IECs in the F_0_ generation based on the filtered high quality methylation values for each covered position ranging from 0 to 1 (*P* = 0.001, multivariate ANOVA). (**d**) Heatmap showing hierarchical clustering of the top 50 differentially methylated genes between F_0_^Ctrl^ and F_0_^DSS^ IECs. The genes along with their genomic coordinates and associated site are also shown on the right column. (**e**) Heatmap showing hierarchical clustering of the top 50 differentially methylated genes between F_1_^Ctrl^ and F_1_^DSS^ mice. The genes along with their genomic coordinates and associated site are also shown on the right column. Blue and red boxes indicate high and low methylation, respectively.

After finding significantly differentially methylated CpG sites in F_0_ sperm (which may shape future F_1_ somatic methylomes) affected by DSS treatment and dysregulated gene expression in their offspring, we analyzed the methylomes of EpCAM^+^ CD45^−^ colonic IECs using RRBS. Despite observing a clear separation of the transcriptomes of IECs between F_1_^Ctrl^ and F_1_^DSS^ mice, MDS analysis of their methylomes (1,037,337 CpG sites identified) did not show a clear separation into two distinct clusters (Fig. 4a). However, comparing the methylomes of F_1_^Ctrl^ and F_1_^DSS^ IECs resulted in 219 differentially methylated CpG sites of which 115 CpGs could be annotated to transcripts and/or promoters (Supplementary Data 6). Consistently, we observed moderate global DNA methylation changes (20% cut-off) in IECs of F_1_^DSS^ mice compared to F_1_^Ctrl^ mice (Fig. 4b) indicating that DSS in F_0_ mice might have a small but significant effect on cytosine methylation in the next generation. For control purposes we also analyzed the methylomes of IECs of the F_0_ generation (904,007 CpG sites identified), which resulted in 2,107 CpG sites that are differentially methylated between F_0_^Ctrl^ and F_0_^DSS^ IECs (353 hypo-methylated and 1,754 hyper-methylated) (Fig. 4c). 866 of these sites could be annotated to transcripts and/or promoters (Supplementary Data 7). The 50 most differentially methylated sites (based on methylation difference) found in IECs of F_0_ and F_1_ mice are shown as heatmaps in Fig. 4d,e, respectively. The proportions of genomic region categories for quality-controlled CpGs in IECs of both generations were similar to what was previously observed in the sperm data (Supplementary Fig. 6a,b).

An overlap of differentially methylated genes in F_0_ and F_1_ IECs resulted in 10 genes (two hypo-methylated and eight hyper-methylated in both generations) (Supplementary Data 8). Four of the ten sites showed a differential DNA methylation within the promoter or the transcription factor-binding region of their annotated genes in F_1_ IECs (associated with genes: *Ick*, *Adcy6*, *Rbfox3* and *Tspear*). Only one differentially methylated site was located at the same inter-genic genomic location and was hypo-methylated in both generations (chr14: 32,700,214).

The DNA methylation pattern of a subset of paternally imprinted genes from the GeneImprint database^55^ was also checked in the epithelial and sperm samples to show that the bisulfite conversion for the RRBS protocol worked. As expected, we observed either no methylation (*Igf2* and *Dio3* genes), complete methylation (*Gpr1*, *Peg10* and *Magi2* genes) or partial methylation (*Mest* and *Plagl1* genes) for these ‘control’ loci in IECs (Supplementary Fig. 7a–d), however, minor shifts were observed in the imprinting patterns of sperm cells (Supplementary Fig. 7e–h).

Finally, the overlap analysis of differential expression and differential DNA methylation in F_1_ IECs resulted in 13 genes (Supplementary Data 9, Supplementary Fig. 8a,b). 8,662 out of 10,000 permutations resulted in an overlap greater or equal to 13 (mean number after 10,000 permutations = 16.984 [3, 37]; Permutation based p-value = 0.86) (data not shown). In summary, these results suggest that DSS-induced colitis in F_0_ males affects the DNA methylation pattern of their germ-line and that intergenerational inheritance of these epigenetic modifications are associated with alterations of the IEC transcriptome and methylome in their offspring.

### Patterns of epigenetic inheritance

**Figure 5:**
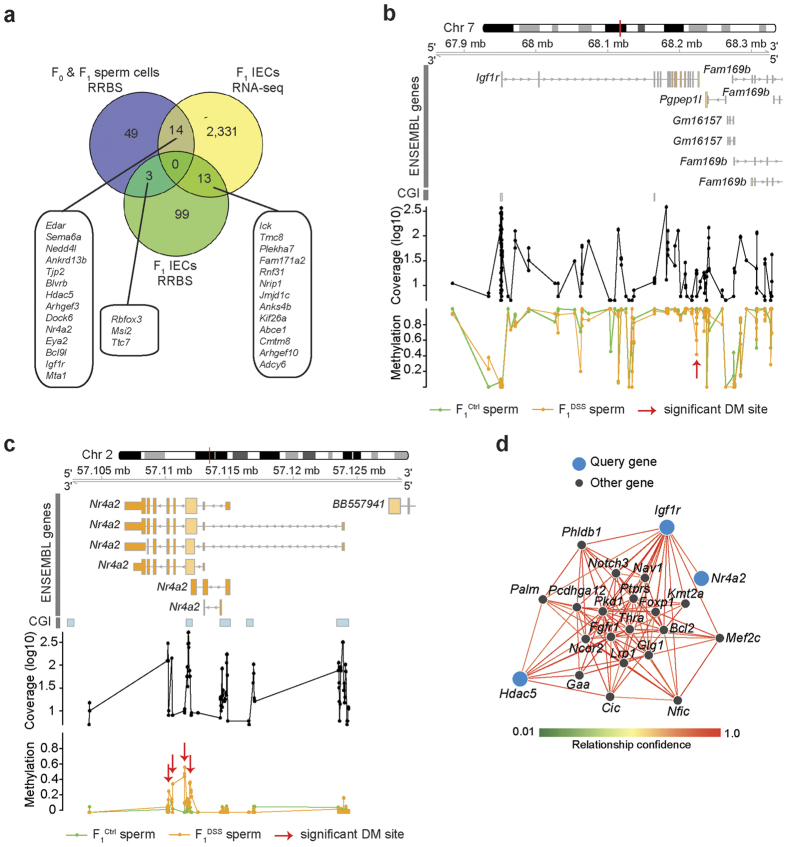
Epigenetic alterations induced by chronic inflammation in the F_0_ generation were stably transmitted to offspring. (**a**) Overlap of differentially methylated genes between sperm samples of F_0_ and F_1_ mice (same direction of effect) with differentially expressed genes in F_1_ IECs as well as with differentially methylated genes in F_1_ IECs. The genes and their associated methylation or expression values are listed in Supplementary Data 9, Supplementary Data 3 and Supplementary Data 7, respectively. (**b,c**) Regional browser-view plots for *Igf1r* (**b**) and *Nr4a2* (**c**) genes that are differentially methylated (DM) in F_0_ and F_1_ sperm samples and differentially expressed in F_1_ IECs. The plots depict the EnsEMBL gene annotations, the log transformed coverage for each quality-controlled site and the median methylation for F_1_^Ctrl^ and F_1_^DSS^ sperm samples. Red arrows indicate significant DM sites in *Igf1r* (**b**) and *Nr4a2* gene (**c**). (**d**) Gene enrichment analysis of the query candidate genes (colored in blue) that remain connected with a relationship confidence of 0.75. Other interacting genes expressed in epithelial tissue are shown as black dots. The displayed network shows predicted functional relationships between the most functionally related genes and the query input genes, which are also analyzed for gene ontology enrichment (Supplementary Data 12). The relationship confidence is supported by several functional genomic and expression datasets. The edges between genes are colored (light red to dark red) by the confidence of the predicted relationship (relationship confidence).

To identify potential candidate genes with DNA methylation patterns that are passed on to the F_1_ generation, we screened for positional overlaps between differentially methylated sites in sperm samples of the F_0_ and F_1_ generation as well as of IECs of the F_1_ generation, which resulted in three inter-generationally inherited genes (*Rbfox3, Msi2* and *Ttc7*) (Supplementary Data 3 and Supplementary Data 7, Fig. 5a). Interestingly, this number is significantly larger than expected by chance in 9,700 out of 10,000 permutations (mean number after 10,000 permutations = 0.677 [0, 6]; Permutation based p-value = 0.03) (Supplementary Fig. 9a). Furthermore, the overlap analysis, comparing differential DNA methylation in sperm samples of the F_0_ and F_1_ generation with the differential expression in F_1_ IECs, suggested 14 different candidate genes that may be epigenetically inherited (Supplementary Data 10, Fig. 5a). Nine genes were negatively regulated of which five were hypo-methylated and up-regulated (*Sema6a, Tjp2, Hdac5, Arhgef3, Dock6*) and four hyper-methylated and down-regulated (*Ankrd13b, Blvrb, Nr4a2, Eya2*). Five were positively regulated with four hypo-methylated and down-regulated (*Edar, Bcl9l, Igf1r, Mta1*) and one hyper-methylated and up-regulated (*Nedd4l*). The regional plots showing the differentially methylated sites for *Igf1r*, *Nr4a2*, *Hdac5* and *Mta1* are shown in Fig. 5b,c and Supplementary Fig. 9b,c.

Next, we compared our F_1_ sperm DNA methylation data with the 17 validated hypo-methylated regions and their associated genes published in the study from Radford *et al*.^37^, who report that *in utero* undernourished pups displayed a lower body weight and suffered from metabolic disorders in their later lives. Interestingly, reporting a similar phenotype of lower body weight herein, we also identified three consistently hypo-methylated genes, i.e. *Asap2* (also significantly up-regulated in F_1_ IECs)*, Wif1* and *Rnf149*. However, the associated CpG sites of these hypo-methylated genes were covered at different locations (Supplementary Data 11).

Finally, we studied the herein-identified candidate genes and their interactions by a systematic functional network analysis (Fig. 5d). Their enriched KEGG (Kyoto Encyclopedia of Genes and Genomes) pathways and Gene ontology (GO) biological process categories are shown in Supplementary Data 12, which highlight pathways involved in notch signaling, melanoma, prostate cancer and transcriptional misregulations affecting several, diverse biological processes like brain development, skeletal muscle development, B cell homeostasis, fear/behavioral defense response, abnormalities with cardiac function, and cell type specific apoptotic process.

## Discussion

To the best of our knowledge, this is the first study investigating environmentally-induced epigenetic changes in the course of DSS-induced colitis in mice. We report that offspring of males that had developed and recovered from chronic DSS colitis (F_1_^DSS^) displayed a lower body weight at baseline conditions compared to offspring of healthy males (F_1_^Ctrl^). F_1_^DSS^ mice were also more susceptible to experimental colitis but did not exhibit spontaneous intestinal inflammation.

We performed RRBS and RNA-Seq to identify differentially methylated genes and its impact on gene expression. Global DNA methylome analysis of sperm cells revealed that the sperm epigenome was affected by prior DSS-induced colitis. Similarly, the methylome and transcriptome of IECs of F_1_^DSS^ mice at baseline showed differentially methylated and expressed genes compared to F_1_^Ctrl^ mice. It had already been reported that unlike any other cell type, massive epigenetic changes take place during spermatogenesis^26^. Environmental triggers may also disturb imprinting of genes that can alter the sperm epigenome, resulting in compromised gene expression in the offspring^56^. Epigenetic modifications may also shape the response and phenotype of offspring to variable environmental conditions^57^,^58^. While several of the identified epigenetic alterations may not affect the expression of the associated genes, they may still be transmitted by a ‘silent carrier’ to the next generations without affecting the gene expression and resulting in an asymptomatic phenotype^59^,^60^.

Notably, we observed moderate global methylation changes (20% cut-off) between IECs of F_1_^Ctrl^ and F_1_^DSS^ mice indicating that DSS might have small but significant effects on cytosine methylation in the next generation. In agreement with our results, Carone *et al*.^30^ reported similar modest effects on cytosine methylations in offspring of males fed a low-protein diet. Strikingly, they found significant alterations in offspring’s gene expression of many hepatic genes involved in lipid and cholesterol biosynthesis^30^.

DSS is a large molecular entity (MW 36–50 kDa) that upon oral ingestion disrupts the intestinal epithelial surface and thereby induces colitis^61^. Since DSS is not present at appreciable levels in the circulation while ingested orally, cell membranes are impermeable to intact molecules, and dextran molecules are not supposed to directly or indirectly introduce methyl groups to DNA, direct DSS effects to the genome appear unlikely, although we cannot formally exclude this possibility. Altogether it is fair to conclude that the biological phenotypes in the F_1_ generation and the changes in the F_0_ sperm methylome, which are – at least partially – also present in F_1_ sperm, may indeed be a consequence of intestinal inflammation.

Notably, almost half of the CpG sites in our data were annotated to regulatory regions of CTCF binding sites supporting the role of chromatin modifications in regulating disease susceptibility and the low weight phenotype. Specifically, CTCF is involved in regulating chromatin structure and plays an important role as a transcriptional repressor of *Igf2* (insulin-like growth factor 2)^62^,^63^,^64^,^65^. Small size at birth and food deprivation due to prenatal exposure to famine has been also linked to hyper-methylation of sensitive loci including the imprinted locus *Igf2*^66^. Similarly, we identified *Igf1r* (insulin-like growth factor 1 receptor) and *Nr4a2/Nurr1* (nuclear receptor subfamily 4, group A, member 2) to be dysregulated in the intestinal epithelium of F_1_^DSS^ mice. Interestingly, *Igf1r* down-regulation has previously been associated with intrauterine and postnatal growth retardation^67^, growth failure, mental retardation and fetal abnormalities^68^. In the herein reported study, *Igf1r* was found to be hypo-methylated and down-regulated in F_0_^DSS^ sperm cells as well as in F_1_^DSS^ IECs and F_1_^DSS^ sperm cells, suggesting a possible link between this gene’s function with the herein identified metabolic phenotype in the F_1_ generation. The other possible epigenetically inherited candidate gene identified in this study, *Nr4a2*, was previously shown to be strongly up-regulated in adipose tissue in human obesity and is also linked to the regulation of body weight^69^,^70^. Furthermore, functional *in vitro* analyses revealed that *Nr4a2* is involved in stress response as well as immune development and function^69^,^70^. Specifically, *Nr4a2* was hyper-methylated and down-regulated in F_1_^DSS^ IECs and F_1_^DSS^ sperm cells, which may possibly represent a link between our metabolic phenotype, differential DNA methylation and consequently modulation of adipose tissue expansion.

Furthermore, our gene set enrichment analysis of differentially expressed genes in F_1_^DSS^ mice collectively suggests a dysregulation of the immune response in these mice, which is in agreement with the increased susceptibility to inflammation detected in F_1_^DSS^ mice.

Altogether, we report that epigenetic modifications acquired during DSS-induced colitis were transmitted to offspring through the paternal germ-line and led to alterations in the methylation pattern of CpG sites situated in genes involved in energy metabolism, which in turn may be associated with a lower body weight and increased susceptibility to colitis in offspring of diseased mice. Moreover, this study provides a list of candidate genes that may contribute to a better understanding of epigenetically modulated pathways during or after chronic intestinal inflammation. As such, our study adds to a growing body of literature^29^,^38^,^39^,^40^,^42^,^43^,^44^,^45^,^46^,^47^,^48^,^49^,^50^,^51^,^52^,^53^, which suggests that paternal lifestyle and pathological conditions (e.g. chronic inflammation) can affect spermatogenesis and can induce intergenerational transmission of epigenetic marks modulating offspring’s metabolism, but also contributing to risk for disease.

## Material and Methods

### Mice

Newborn male and female C57Bl/6(N) littermate mice were purchased from Charles River Laboratories. For all mice used in this study it was requested that n ≥ 6 mice of the same gender came from the same litter. Mice were maintained under specific pathogen free (SPF) conditions and were kept with a strict 12-hour light/dark cycle at the ZVTA (Medical University Innsbruck). All mice received the same diet and were fed according to a strict schedule. At 13 weeks of age male littermates were mated overnight with female littermates. After mating, males were single housed and were allowed to refill their sperm reservoir for 4 days, before they were sacrificed at 13.5 weeks of age. The Austrian Ministry of Science and Research has approved all mouse protocols and all experiments were performed in accordance with institutional guidelines.

### Induction of chronic colitis by dextran sodium sulphate (DSS)

Chronic experimental colitis was induced in 5-week-old male C57Bl/6(N) littermates (F_0_^DSS^) by administration of three cycles of 2% dextran sodium sulphate (DSS Reagent grade, MW 36–50 kDa, MP Biomedicals, LLC) in their drinking water for 5 consecutive days, followed by a 14-day tap water period each. In the meantime normal drinking water was administered to the other half of the litter (F_0_^Ctrl^). During DSS treatment the disease activity index was determined daily by combining scores of weight loss, consistency of stool and rectal bleeding^71^. 9 days after the last DSS administration (day 55 of colitis model), F_0_^DSS^ and F_0_^Ctrl^ male littermates were mated overnight with littermate females.

### Inflammation susceptibility test by induction of an acute DSS colitis

Acute colitis was induced in 7-week-old offspring of F_0_^Ctrl^ and F_0_^DSS^ mice by administration of 3.5% DSS in their drinking water for 6 consecutive days. During DSS treatment the disease activity index was determined daily by combining scores of weight loss, consistency of stool and rectal bleeding. After sacrifice, histological evaluations and length measurements were performed on extracted colons.

### Histology

Formalin-fixed and paraffin-embedded intestines were sectioned and stained with hematoxylin & eosin (H&E) as described previously^72^. Baseline inflammation in the small intestine was assessed on H&E stained sections using a score, which considers four criteria: mononuclear cell infiltrate (0–3), crypt hyperplasia (0–3), epithelial injury (0–3) and polymorphonuclear cell infiltrates (1–3). As described previously^72^, the area involved was incorporated in the score by adding 1 for <10%, 2 for 10–25%, 3 for 25–50% and 4 for >50% involvement. Histological scoring of DSS treated mice was performed as described previously^71^.

### Isolation of intestinal epithelial cells (IECs)

Mice were euthanized at 13 weeks of age and the middle part of the colon was used for the isolation of intestinal epithelial cells (IECs). The colon parts were washed with ice-cold PBS after cut open longitudinally. Mucus was removed by shaking the intestine in 1x HBSS containing 10% FCS, 1 mM DTT, 2 mM EDTA for 10 min at 37 °C. Then the DTT concentration was increased to 2 mM and shaken another 10 min at 37 °C. After moderate vortexing for 3 × 4 min in 1x HBSS containing 10% FCS and 1 mM EDTA, intestinal epithelial cells (IECs) were poured through 70 μm cell strainer (BD Bioscience), centrifuged at 1,500 rpm for 10 min and resuspended in PBS for subsequent FACS analysis.

### Sperm cell isolation

Sperm cells from sacrificed mice were isolated from the caudal epididymis and *Vas deferens* as described previously^30^. Briefly, sperm was allowed to release from punctured and incised caudal epididymis and *Vas deferens* by incubation for 30 min at 37 °C in M2 medium (Sigma-Aldrich). Released sperm was collected and determined by microscopy for motility and purity. After RNA isolation qRT-PCR of epididymis-specific (*Myh11, Actb*) and sperm-specific genes (*Smcp*, *Odf1*) was performed to assess purity of sperm preparations.

### Hematocrit measurement

Mice were anesthetized using a ketamine/xylazine mixture and blood samples were taken from the heart using a heparinized needle and syringe. Hematocrit levels in the whole blood were measured with a veterinary animal blood counter (Scil vet abc, HORIBA Medical).

### RNA sequencing (RNA-Seq)

RNA libraries were prepared using the TruSeq RNA Sample Preparation Kit v2 (Illumina) according to manufacturer’s instructions. Libraries were analyzed with an Agilent 2100 Bioanalyzer using the Agilent DNA 1000 Kit. All samples were sequenced using an Illumina HiSeq 2000 platform (Illumina) at an average of 40 million paired-end 100 bp reads (Supplementary Data 13). Analytical details are provided in Supplementary Methods.

### Reduced Representation Bisulfite Sequencing (RRBS)

RRBS libraries of genomic DNA isolated from sperm cells and FAC-sorted EpCAM^+^ CD45^−^ colonic IECs were prepared as described by Boyle and colleagues^73^ and Smallwood and Kelsey^74^ using 5mC sequencing adapters (Illumina) and PE1.0 and PE2.0 primers (Illumina) for single-sample sequencing and TruSeq adapters and primers (Illumina) for the multiplexed sequencing approach. The final amplification was performed in accordance to the conditions described by Gu and colleagues^75^. All libraries were sequenced using a HiSeq 2500 platform (Illumina) at an average of 127 million single-end 50 bp reads (Supplementary Data 14). Additional details and analytical methods are provided in Supplementary Methods.

### Statistical analysis of *in vivo* model data

Data are presented as mean ± standard error of the mean (SEM). Statistical analysis was performed using Microsoft Excel (Microsoft) and GraphPad Prism 6 (GraphPad Software). If not stated otherwise, statistical significance was calculated using a two-tailed Student’s *t-*test or a Mann-Whitney *U* test and *P *< 0.05 was considered significant. Where more than two groups were compared, One-way ANOVA with Bonferroni’s post-hoc testing or Kruskal-Wallis-tests were performed.

## Additional Information

**How to cite this article**: Tschurtschenthaler, M. *et al*. Paternal chronic colitis causes epigenetic inheritance of susceptibility to colitis. *Sci. Rep.* **6**, 31640; doi: 10.1038/srep31640 (2016).

## Supplementary Material

Supplementary Information

Supplementary Data
