# Supplementary material for: Paternal chronic colitis causes epigenetic inheritance of susceptibility to colitis
Source: Sci Rep. 2016 Aug 19;6:31640. doi: 10.1038/srep31640 (PMC4990911; doi:10.1038/srep31640)

Supplementary Information for

Paternal chronic colitis causes epigenetic inheritance of susceptibility to colitis

Markus Tschurtschenthaler, Priyadarshini Kachroo, Femke-Anouska Heinsen, Timon Erik Adolph, Malte Christoph Rühlemann, Johanna Klughammer, Felix Albert Offner, Ole Ammerpohl, Felix Krueger, Sébastien Smallwood, Silke Szymczak, Arthur Kaser, Andre Franke

Inventory

Supplementary Materials and Methods

Supplementary Figure legends

Supplementary Dataset legends

Supplementary References

Supplementary Materials and Methods

Flow cytometry (FACS). Single cell suspensions were incubated with the Fc-Block CD16/CD32 (BD Bioscience) for 10 min on ice. Antibody staining using antibodies against CD45 (FITC conjugated, Biolegend) and CD326 (EpCAM; PE conjugated, eBioscience) was performed according to manufacturer's instruction. DAPI (eBioscience) was used as a live vs. dead discriminator. Samples were analyzed on a BD LSR Fortessa cell analyzer or with a FACSAria flow cytometer (BD Biosciences) and $EpCAM^{+}$ $CD45^{-}$ epithelial cells were sorted and isolated.

DNA and RNA isolation. Genomic DNA and total RNA were extracted from freshly isolated and unfixed $EpCAM^{+}$ $CD45^{-}$ intestinal epithelial cells (IECs) and sperm cells using the AllPrep DNA/RNA Mini or Micro Kit (Qiagen) according to manufacturer's instructions. Quality and quantity of DNA and RNA was assessed with a Qubit fluorometer (Invitrogen) using Quant-iT PicoGreen dsDNA Kit and Quant-iT RNA Assay Kit, respectively.

RNA-Seq data pre-processing and analysis. Removal of bad quality reads was carried out using Illumina's CASAVA filter version 0.1 and quality checks were performed with FastQC v0.10.1. Sequence reads were aligned to the UCSC mm10 reference genome from UCSC, using Tophat2 [1] and Bowtie2 version 2.0.0-beta6 [2]. Gene expression was quantified as read counts calculated using the python tool HTSeq based on UCSC RefSeq gene annotations [3]. Genes with at least 1 count per million (cpm) in at least five samples were retained for further analysis. To account for the different sequencing depths between samples, TMM normalization was applied and differential expression analysis was performed as implemented in the Bioconductor package edgeR [4]. Benjamini–Hochberg correction was used to adjust for

multiple testing. Overrepresentation of gene ontology categories regarding biological processes was analyzed using the Bioconductor package goseq. The hyper-geometric test was applied since no gene length biases were detected with all analyzed genes as reference. The list of significantly expressed genes was further filtered to genes with an absolute log-transformed fold change >1. Significant categories and corresponding genes were visualized using the Bioconductor package GeneAnswers [5].

Reduced Representation Bisulfite Sequencing (RRBS). 100-250 ng gDNA were subjected to MspI digest (37°C for 5 h), DNA end repair and A-tailing as described by Boyle and colleagues [6] with subsequent heat inactivation as described by Smallwood and Kelsey [7] and AMPure XP beads purification (Beckmann Coulter) according to the manufacturer's instructions.. Adapter ligation was performed at 16°C overnight [6] and subsequent heat inactivation at 65°C for 20 min [7] by using T4 DNA ligase (Thermo Fisher) and Illumina 5mC sequencing adapters for single-sample sequencing and Illumina TruSeq adapters for the multiplexed sequencing approach, respectively. After AMPure XP beads purification (Beckmann Coulter) samples were bisulfite converted using one-step modification of the Imprint DNA Modification Kit (Sigma). Final amplification of converted samples was performed by using PfuTurbo Cx Hotstart DNA Polymerase (Agilent) and Illumina primers PE1.0 and PE2.0 for single-samples sequencing and Illumina TruSeq primers for multiplexed sequencing [6] with PCR conditions as described in Gu and colleagues [8]. PCR products were purified using AMPure XP beads (Beckmann Coulter) and indexed samples were pooled equimolar into pools of six. Libraries were analyzed with Agilent 2100 Bioanalyzer using the Agilent High Sensitivity DNA Kit and concentration was measured using Quant-iT DNA BR Assay Kit (Life Technologies). All sperm and epithelial samples were sequenced using an Illumina HiSeq 2500 platform (Illumina, San Diego, CA) at an average of 127 million single-end 50 bp reads (**Supplementary Data 14**).

RRBS data pre-processing and analysis. Illumina CASAVA filter version 0.1 and FastQC v0.10.1 were used for removing bad quality reads and for quality control, respectively. Adapter or primer contamination was removed using cutadapt. Alignment to bisulfite converted mm10 reference genome from UCSC and methylation calling was performed using Bismark v0.7.12 [9,10]. The R-package RnBeads v0.99.16 [11] was employed for additional filtering steps, and differential methylation analysis. Low quality sites present in less than 50% of the samples and sites with extremely large total coverage (total coverage > 99% quantile of all sites) were removed and only autosomal sites were retained in the study. Two samples were removed from the analysis due to gender mix-up or a too low number of covered CpG sites. Site-based differential methylation analysis was performed applying the limma method [12] to mouse litters, library preparation batches, concentration, sequencing batches, gender as well as covariates wherever applicable.

For our analysis, a site was called differentially methylated (DM) if the raw p-value was smaller than 0.05 and the absolute mean methylation difference was at least 0.20. DM sites were categorized as hyper- or hypo-methylated if they showed higher or lower methylation ratios in DSS-treated or offspring mice compared to control mice, respectively. A gene was annotated or called if at least one CpG site within the gene sequence or promoter was differentially methylated. A site can be located in gene body, promoter, intron or exon, therefore all sites irrespective of their location, whether downstream or upstream of the associated region were retained for further downstream analyses. Additionally, if more than 1 site was associated with a gene, the site with the largest methylation difference was selected for a particular gene.

Direction of the biological effect had to be the same to classify it as an ‘overlap’. For differential expression, transcripts had to be either up- or down-regulated in both the F_0

generation and their offspring. For differential methylation, CpG sites had to be either hypo- or hyper-methylated in both F_0 generation and their offspring in every analyzed tissue.
To check if the observed number of overlapping genes is different from the expected number under independence of findings in the F_0 and F_1 generation, we performed the following permutation test. Observed p-values were randomly shuffled across all filtered CpG sites in F_0 and F_1 generation separately and the number of differentially methylated genes detected in both generations was calculated using the same definition described above. This process was repeated 10,000 times to estimate the mean number of overlapping genes and the p-value as the number of permutations with an overlap larger or equal than the observed number of overlaps.

Genomic annotation of differentially methylated sites. Differentially methylated CpG sites were annotated with respect to known CpG islands (CGIs), genes and regulatory regions. CGI definitions were downloaded from UCSC and CGI shores were defined as regions outside CGIs but within 2 kbp of any CGI and CGI shelves as regions within 2 kbp from a CGI shore. Information about genes including transcript, introns, exons, 3' UTR and 5' UTR was extracted from Bioconductor packages Mus.musculus and TxDb.Mmusculus.UCSC.mm10.knownGene version 3.1.2. Promoters were defined as regions 1 kbp upstream of a transcription start site (TSS). Regulatory information including enhancers and transcription factor binding sites was downloaded from EnsEMBL build GRCm38.p4 (Genome Reference Consortium Mouse Reference 38).
Functional annotation of differentially methylated regions (DMRs) was performed using the gene enrichment tool GREAT [13]. To reduce the number of false positives, basal plus extension rule was applied including default parameters. Target genes can be regulated by regulatory elements or domains that are located over one million bases away from them [14] as defined by the 'basal plus extension rule' of GREAT to even detect long-range interactions.

Correlation of methylation data with gene expression. For each gene that is either differentially methylated or has a differentially methylated site in its promoter region, the CpG site with the strongest absolute methylation difference was selected and correlation with the corresponding normalized gene expression values was estimated using Pearson's correlation coefficient.

Network analysis. Functional network analysis and gene enrichment was performed using the FNMT web tool (functional networks of tissues in mouse) [15] using 'Colon' as the reference tissue and our candidate genes as input (**Supplementary Data 10**). Minimum relationship confidence of 0.75 was used to observe major interacting and co-expressing genes.

Supplementary Figure Legends

Supplementary Figure 1. Experimental model to study epigenetic alterations induced by chronic inflammation and their potential of intergenerational inheritance. (**a**) C57Bl/6 littermate males (6 pups in one litter) and females (6 pups in one litter) were purchased from Charles River. At 5 weeks of age litters with male pups were split into two groups: F_0^{Ctrl} (kept on water) and F_0^{DSS} (undergoing DSS treatment to develop chronic colitis). Litters consisting of female pups were all kept on normal drinking water. (**b**) Representative weight change of 5 week-old male littermate mice in the course of chronic colitis using DSS in the drinking water (F_0^{DSS}) compared to littermate mice that were meanwhile kept on normal drinking water (F_0^{Ctrl}). F_0^{Ctrl}, n=3; F_0^{DSS}, n=3; Mean ± SEM. (**c**) Representative H&E stainings of the distal colon of F_0^{DSS} mice after healing colitis for 18 days after the last DSS cycle in the model of chronic DSS-induced colitis (scale bars, 200µm). (**d**) DSS subscores (left) and the resulting cumulative score (right) of the distal colon of F_0^{DSS} after recovering from colitis for 18 days after the last DSS cycle. F_0^{DSS}, n=5-6 from two representative litters; data are presented as Median. (**e**) Colon length of F_0^{DSS} mice after healing chronic DSS-induced colitis for 18 days compared to littermate mice kept on normal drinking water (F_0^{Ctrl}). F_0^{Ctrl}, n=21; F_0^{DSS}, n=15; Mean ± SEM; Unpaired, two-tailed Student's t-test. (**f**) F_0^{Ctrl} and F_0^{DSS} male littermates were crossed with female littermates in order to generate F_1 offspring.

Supplementary Figure 2. Increased susceptibility to DSS-induced colitis in offspring of mice with chronic experimental colitis. (**a,b**) DSS subscores (left) and the resulting cumulative score (right) of the proximal colon of F_1^{Ctrl} and F_1^{DSS} females (**a**) and males (**b**) after induction of an acute colitis by adding 3.5% DSS in the drinking water of mice of both groups (F_1 DSS susceptibility test). F_1^{Ctrl} females, n=9; F_1^{DSS} females, n=9; F_1^{Ctrl} males, n=6; F_1^{DSS} males, n=3; Mean ± SEM; Mann–Whitney *U*-test. (**c-d**) Blood hematocrit values of

F_1^{Ctrl} and F_1^{DSS} mice after 3.5% DSS treatment shown in **Fig. 1e** separated according to gender: females (**c**) and males (**d**). F_1^{Ctrl} females, n=11; F_1^{DSS} females, n=8; F_1^{Ctrl} males, n=6; F_1^{DSS} males, n=3; Mean ± SEM; Unpaired, 1-tailed Student's t-test. (**e-f**) DSS subscores (left) and the resulting cumulative score (right) of the distal colon of F_1^{Ctrl} and F_1^{DSS} females (**e**) and males (**f**) after induction of an acute colitis by adding 3.5% DSS in the drinking water of mice of both groups. F_1^{Ctrl} females, n=10; F_1^{DSS} females, n=9; F_1^{Ctrl} males, n=6; F_1^{DSS} males, n=3; Mean ± SEM; Mann–Whitney *U*-test. (**g**) Body weight reduction (in %) of 7 week-old males and females that are offspring of either F_0^{Ctrl} males (F_1^{Ctrl}) or F_0^{DSS} males (F_1^{DSS}) in the course of an acute colitis induced by 3.5% DSS in the drinking water of both groups for 6 days. F_1^{Ctrl} females, n=11; F_1^{DSS} females, n=9; F_1^{Ctrl} males, n=6; F_1^{DSS} males, n=3; Mean ± SEM. (**h**) Colon length of F_1^{Ctrl} and F_1^{DSS} mice (female and males) that underwent a 3.5% DSS colitis model for 6 days. F_1^{Ctrl} females, n=9; F_1^{DSS} females, n=9; Mean ± SEM; Unpaired, two-tailed Student's t-test.

Supplementary Figure 3. Quality and purity checks of samples analyzed in this study. (**a**) FACS gating strategy used for sorting $EpCAM^+$ $CD45^-$ colonic intestinal epithelial cells (IECs). (**b**) Quality control of normalized counts for RNA-Seq samples. The y-axis shows the log transformed normalized counts for all IEC samples (F_0^{Ctrl} and F_1^{Ctrl} samples shown in green and F_0^{DSS} and F_1^{DSS} samples shown in orange) shown along the x-axis (**c-e**) Normalized counts for genes specifically expressed in epithelia (*Cdh1*, *Actb, Mhy11*), but not in isolated sperm cells, showing purity of isolated samples and no contaminations with surrounding tissues (i.e. epididymis). (**e-g**) Normalized counts for genes specifically expressed in sperm cells (*Odf1*, *Smpc1*), but not in epithelia (i.e. epididymis), showing purity of isolated samples. (**h**) Expression of the epididymis-specific gene *Myh11* as well as of the sperm-specific genes *Odf1* and *Smcp* in representative sperm samples used for RRBS as measured by qRT-PCR.

Supplementary Figure 4. Quality control of samples and genomic coverage annotation of methylated sites in sperm samples. (**a**) MDS analysis of all the filtered and quality controlled CpG sites identified in sperm and epithelium samples (F_0 and F_1) shows a clear separation of both cell types in two clusters. (**b,c**) Genomic coverage annotation of the quality controlled CpG sites in sperm samples of F_0^{Ctrl} and F_0^{DSS} mice (**b**) as well as of F_1^{Ctrl} and F_1^{DSS} mice (**c**) to genic regions, CGIs and regulatory regions. .

Supplementary Figure 5. Differentially methylated genes are overlapping between sperm samples of the F_0 and F_1 generation. (**a**) Permutation analysis was performed to determine whether the observed differential methylation seen in sperm (F_0 and F_1) is larger than expected by random chance. The p-value was estimated as the number of permutations with an overlap larger or equal than the observed number of overlaps (66) represented by the red line. This number is significantly larger than expected by chance in all 10,000 permutations represented by the histogram and the blue line (mean number after 10,000 permutations = 15.518 [3, 30]; Permutation based p-value = 0). (**b,c**) Heatmap of 66 differentially methylated overlapping genes comparing sperm samples of mice of the F_0 generation with mice of the F_1 generation, shows hierarchical clustering between F_0^{Ctrl} and F_0^{DSS} sperms (**b**) as well as between F_1^{Ctrl} and F_1^{DSS} sperms (**c**).

Supplementary Figure 6. Genomic coverage annotation of methylated sites in IECs. (a,b) Genomic coverage annotation of quality controlled CpG sites in IECs of F_0^{Ctrl} and F_0^{DSS} mice (**a**) as well as F_1^{Ctrl} and F_1^{DSS} mice (**b**).

Supplementary Figure 7. Patterns of imprinting in epithelial and sperm samples of mice of the F_0 and F_1 generation. (**a-h**) A few paternally imprinted genes were selected from the GeneImprint database and the housekeeping gene *Actb* was used as a negative control. Y-axis

shows the median methylation values for all the sites covered in the selected genes across x-axis. For IEC samples (**a-d**), the methylation value fluctuates between 0%, 50% or 100% for all genes as expected for imprinted genes. However, for sperm samples (**e-h**), the imprinting patterns of methylation for the selected genes were slightly perturbed.

Supplementary Figure 8. Differential methylation and differential expression in F_1 IECs. (**a,b**) Heatmaps of F_1^{Ctrl} and F_1^{DSS} IECs showing hierarchical clustering of 13 differentially expressed (**a**) and methylated genes (**b**), respectively.

Supplementary Figure 9 | Patterns of epigenetic inheritance – suggested candidate genes. (**a**) Permutation analysis (see Supplementary Material and Methods) was performed to determine whether the observed differential methylation seen in sperm of both F_0 and F_1 generation and in F_1 IECs is larger than expected by random chance. The p-value was estimated as the number of permutations with an overlap larger or equal than the observed number of overlaps (3) represented by the red line. This number is significantly larger than expected by chance in 9,700 out of 10,000 permutations represented by the histogram and the blue line (mean number after 10,000 permutations = 0.677 [0, 6]; Permutation based p-value = 0.03). (**b,c**) Regional browser-view plots for *Hdac5* (**b**) and *Mta1* (**c**) genes that are differentially methylated (DM) in F_0 and F_1 sperm cells and differentially expressed in F_1 IECs, respectively. The plots show the EnsEMBL gene annotations, coverage (log10) distribution of quality-controlled sites, and their median methylation values (hypo-methylated or hyper-methylated). The red arrows indicate sites of *Hdac5*:102,204,587 (**b**) and *Mta1*:113,132,074 (**c**) that are significantly differentially methylated between F_1^{Ctrl} and F_1^{DSS} sperm samples.

Supplementary Data legends

Supplementary Data 1. Differentially methylated sites (RRBS) in sperm samples of F_0^{DSS} and F_0^{Ctrl} mice annotated either to transcript or promoter regions (methylation difference cut off 0.05 and p-value < 0.05). The methylation (AQ-BP) and coverage values (BQ-CP) for each sample, mean methylation (D-E) and mean coverage (Q-R) for each group and the annotations for each differentially methylated CpG site (Y-AP) are listed.

Supplementary Data 2. Differentially methylated sites (RRBS) in sperm samples of F_1^{DSS} and F_1^{Ctrl} mice annotated either to transcript or promoter regions (methylation difference cut off 0.05 and p-value < 0.05). The methylation (AQ-BE) and coverage values (BF-BT) for each sample, mean methylation (D-E) and mean coverage (Q-R) for each group and the annotations for each differentially methylated CpG site (Y-AP) are listed.

Supplementary Data 3. Overlap of differentially methylated genes (RRBS) in F_0 sperm samples with differentially methylated genes (RRBS) in F_1 sperm samples.

Supplementary Data 4. Differentially expressed genes (RNA-Seq) in IEC samples of F_0^{DSS} and F_0^{Ctrl} mice (adjusted p-value < 0.05). The normalized gene expression counts for each sample (G-O) are indicated.

Supplementary Data 5. Differentially expressed genes (RNA-Seq) in IEC samples of F_1^{DSS} and F_1^{Ctrl} mice (adjusted p-value < 0.05). The normalized gene expression counts for each sample (G-AC) are indicated.

Supplementary Data 6. Differentially methylated sites (RRBS) in IEC samples of F_0^{DSS} and F_0^{Ctrl} mice annotated either to transcript or promoter regions (methylation difference cut off 0.05 and p-value < 0.05). The methylation (AQ-BQ) and coverage values (BR-CR) for each sample, mean methylation (D-E) and mean coverage (Q-R) for each group and the annotations for each differentially methylated CpG site (Y-AP) are listed.

Supplementary Data 7. Differentially methylated sites (RRBS) in IEC samples of F_1^{DSS} and F_1^{Ctrl} mice annotated either to transcript or promoter regions (methylation difference cut off 0.05 and p-value < 0.05). The methylation (AQ-BL) and coverage values (BM-CH) for each sample, mean methylation (D-E) and mean coverage (Q-R) for each group and the annotations for each differentially methylated CpG site (Y-AP) are listed.

Supplementary Data 8. Overlap of differentially methylated genes (RRBS) in F_0 IECs with differentially methylated genes (RRBS) in F_1 IECs.

Supplementary Data 9. Overlap of differential methylated genes (RRBS) in F_1 IECs with differential expressed genes (RNA-Seq) found in F_1 IECs along with their correlations.

Supplementary Data 10. Potential candidates for trans-generational inheritance: Overlap of differentially methylated genes in F_0 and F_1 sperm samples with differentially expressed genes in F_1 IECs.

Supplementary Data 11. Overlap between the herein identified differentially methylated regions in F_1 sperm samples (RRBS) with differentially methylated regions identified in the study from Radford and colleagues [16].

Supplementary Data 12. Functional network analysis for the connected genes showing enriched Gene ontology (GO) terms and KEGG (Kyoto Encyclopedia of Genes and Genomes) pathways.

Supplementary Data 13. RNA-Seq statistics of all analyzed F_0 and F_1 IEC samples.

Supplementary Data 14. RRBS statistics of all analyzed F_0 and F_1 samples from sperm cells and IECs.

Supplementary References

1 Trapnell, C., Pachter, L. & Salzberg, S. L. TopHat: discovering splice junctions with RNA-Seq. *Bioinformatics* **25**, 1105-1111, doi:10.1093/bioinformatics/btp120 (2009).

2 Langmead, B. & Salzberg, S. L. Fast gapped-read alignment with Bowtie 2. *Nat Methods* **9**, 357-359, doi:10.1038/nmeth.1923 (2012).

3 Anders, S., Pyl, P. T. & Huber, W. HTSeq--a Python framework to work with high-throughput sequencing data. *Bioinformatics* **31**, 166-169, doi:10.1093/bioinformatics/btu638 (2015).

4 Robinson, M. D., McCarthy, D. J. & Smyth, G. K. edgeR: a Bioconductor package for differential expression analysis of digital gene expression data. *Bioinformatics* **26**, 139-140, doi:10.1093/bioinformatics/btp616 (2010).

5 Huang, L. *et al.* GeneAnswers: Integrated Interpretation of Genes. R package version 2.10.0. (2014).

6 Boyle, P. *et al.* Gel-free multiplexed reduced representation bisulfite sequencing for large-scale DNA methylation profiling. *Genome Biol* **13**, R92, doi:10.1186/gb-2012-13-10-r92 (2012).

7 Smallwood, S. A. & Kelsey, G. Genome-wide analysis of DNA methylation in low cell numbers by reduced representation bisulfite sequencing. *Methods Mol Biol* **925**, 187-197, doi:10.1007/978-1-62703-011-3_12 (2012).

8 Gu, H. *et al.* Preparation of reduced representation bisulfite sequencing libraries for genome-scale DNA methylation profiling. *Nat Protoc* **6**, 468-481, doi:10.1038/nprot.2010.190 (2011).

9 Krueger, F. & Andrews, S. R. Bismark: a flexible aligner and methylation caller for Bisulfite-Seq applications. *Bioinformatics* **27**, 1571-1572, doi:10.1093/bioinformatics/btr167 (2011).

10 Krueger, F., Kreck, B., Franke, A. & Andrews, S. R. DNA methylome analysis using short bisulfite sequencing data. *Nat Methods* **9**, 145-151, doi:10.1038/nmeth.1828 (2012).

11 Assenov, Y. *et al.* Comprehensive analysis of DNA methylation data with RnBeads. *Nat Methods* **11**, 1138-1140, doi:10.1038/nmeth.3115 (2014).

12 Ritchie, M. E. *et al.* limma powers differential expression analyses for RNA-sequencing and microarray studies. *Nucleic Acids Res* **43**, e47, doi:10.1093/nar/gkv007 (2015).

13 McLean, C. Y. *et al.* GREAT improves functional interpretation of cis-regulatory regions. *Nat Biotechnol* **28**, 495-501, doi:10.1038/nbt.1630 (2010).

14 Lettice, L. A. *et al.* A long-range Shh enhancer regulates expression in the developing limb and fin and is associated with preaxial polydactyly. *Hum Mol Genet* **12**, 1725-1735 (2003).

15 Goya, J. *et al.* FNTM: a server for predicting functional networks of tissues in mouse. *Nucleic Acids Res*, doi:10.1093/nar/gkv443 (2015).

16 Radford, E. J. *et al.* In utero effects. In utero undernourishment perturbs the adult sperm methylome and intergenerational metabolism. *Science* **345**, 1255903, doi:10.1126/science.1255903 (2014).

Supplementary Figure 1

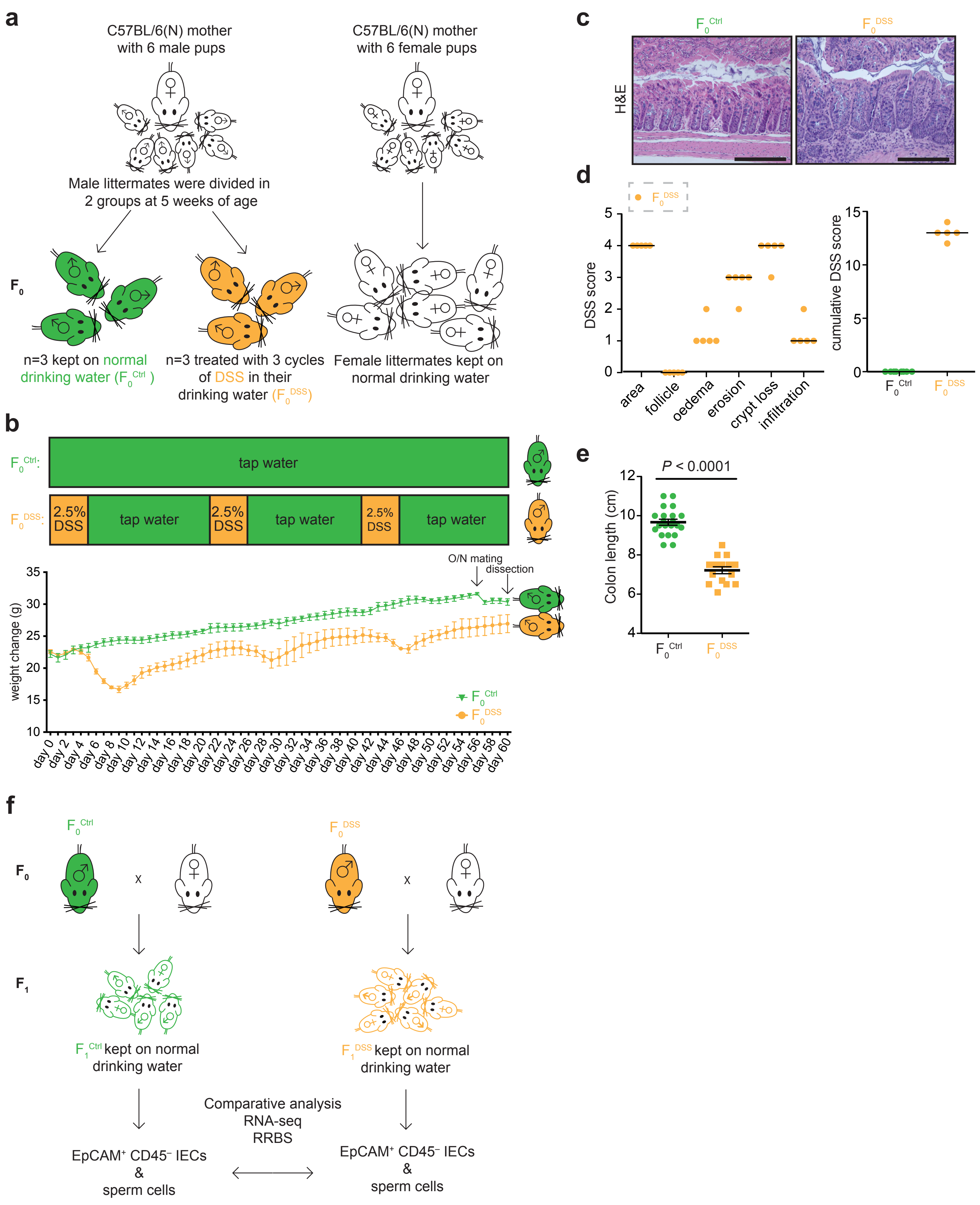

Supplementary Figure 2

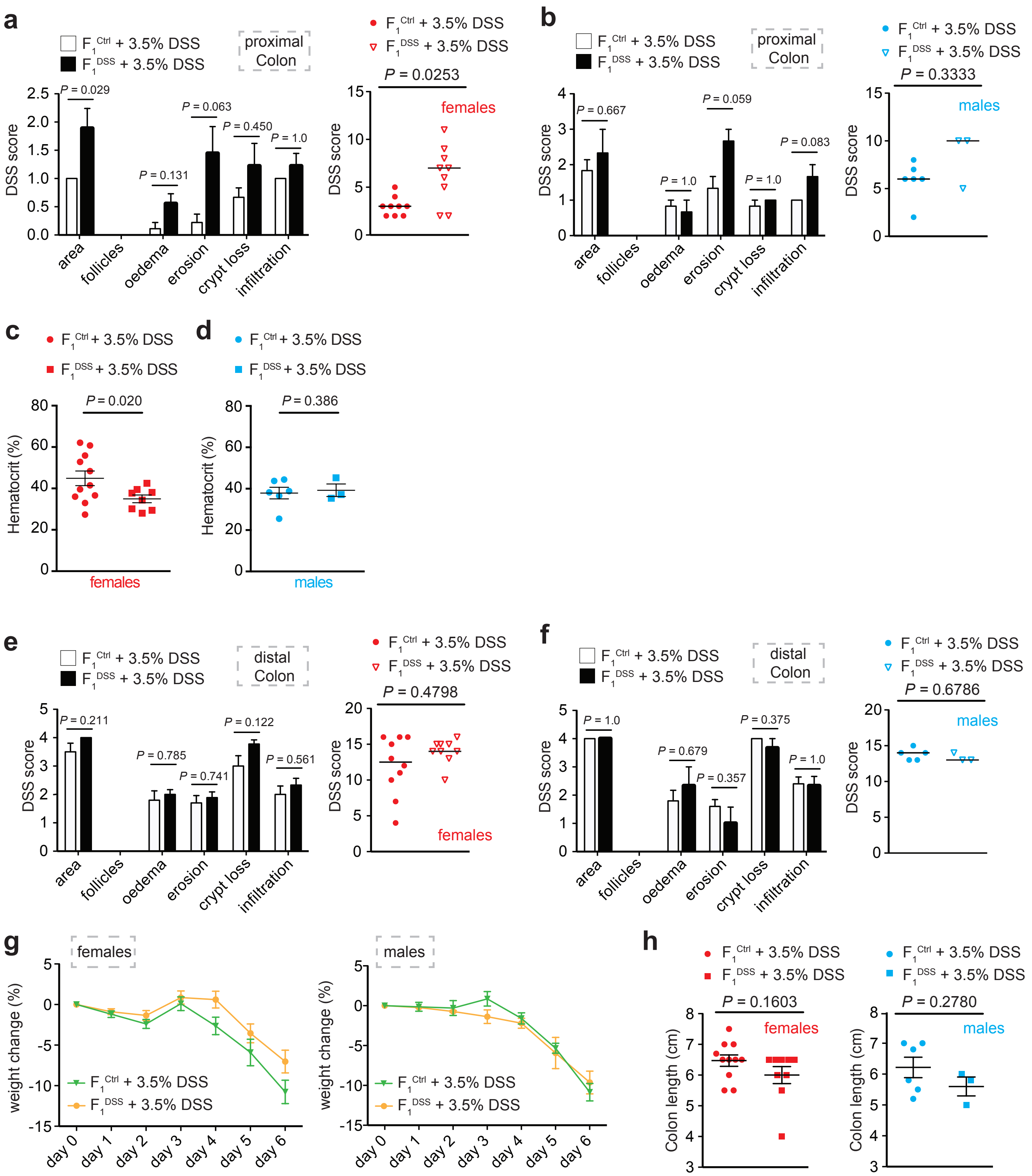

Supplementary Figure 3

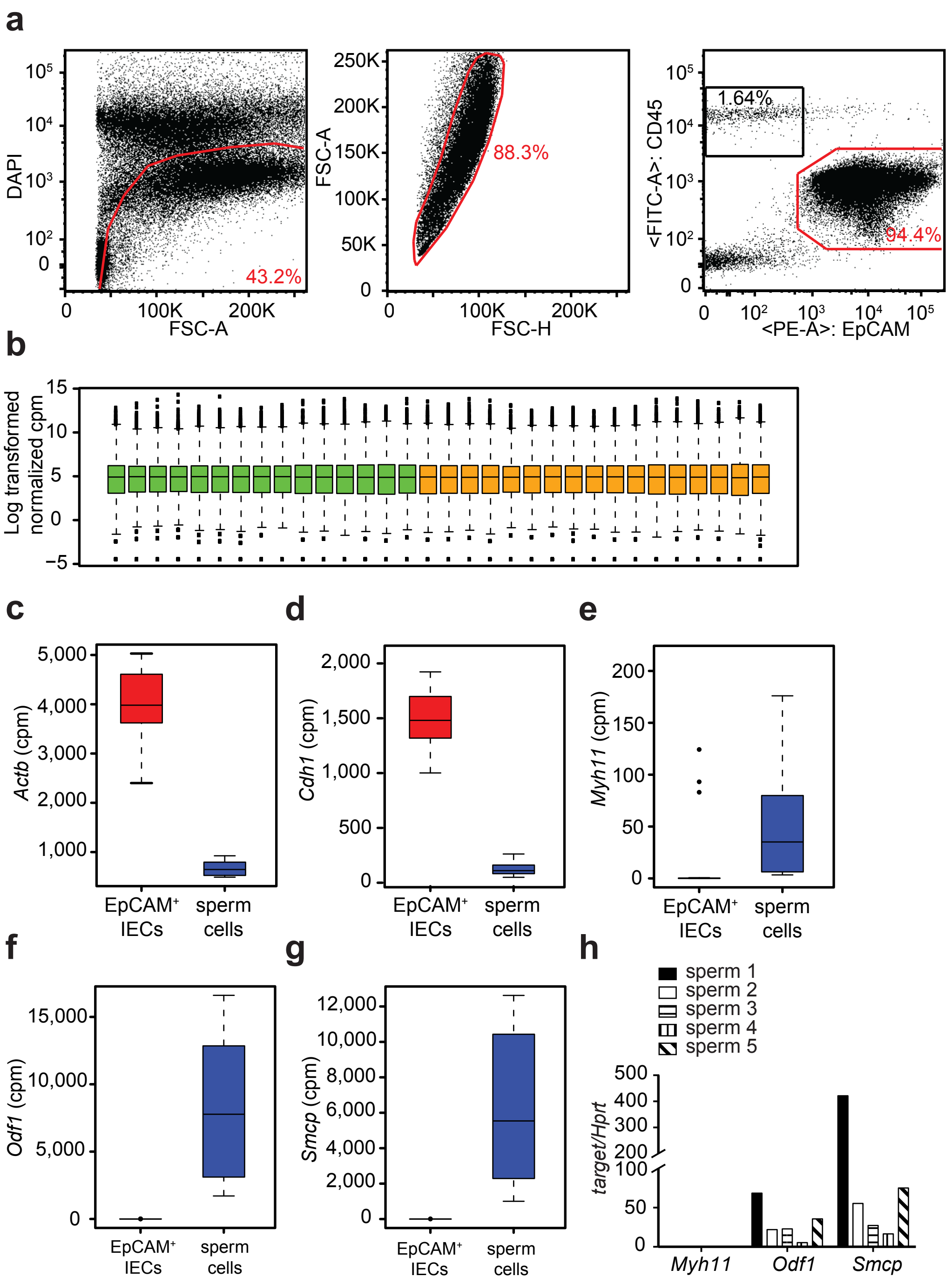

Supplementary Figure 4

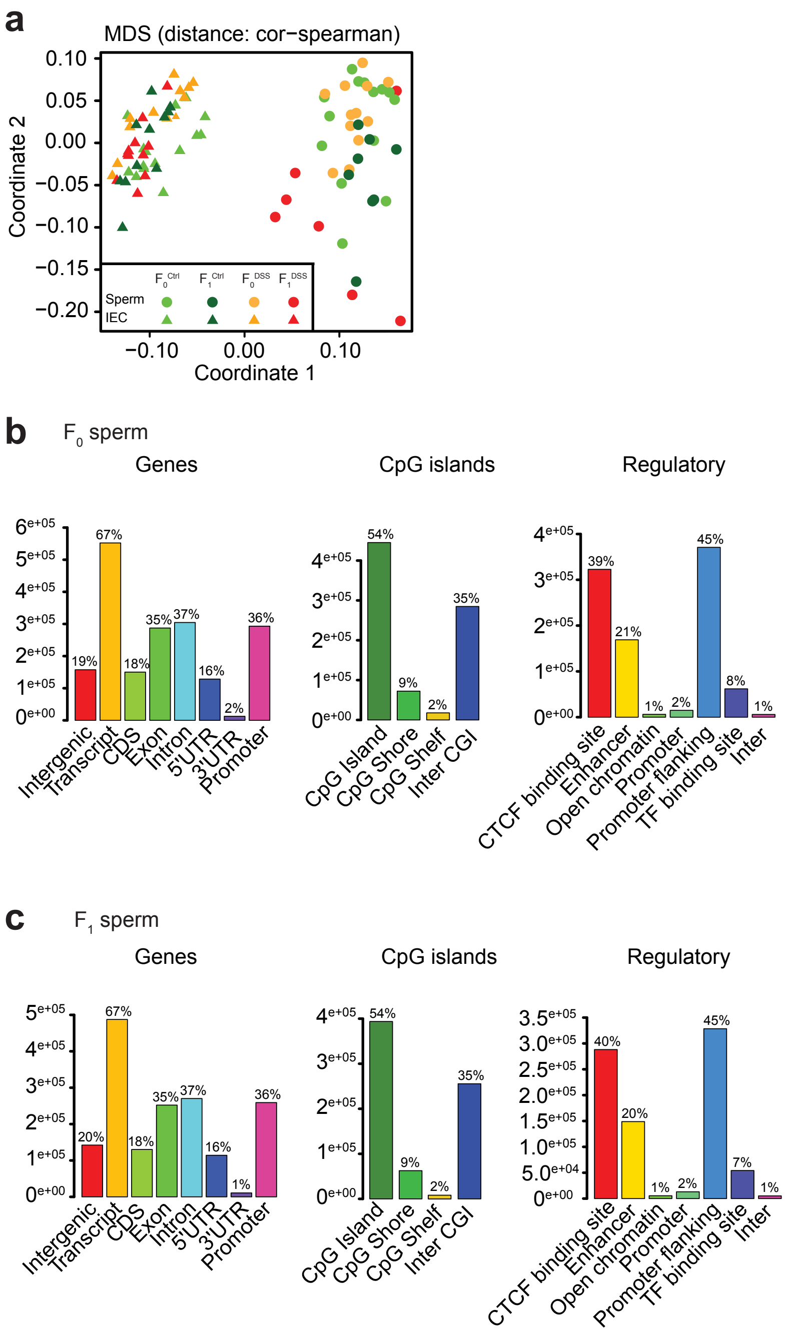

Supplementary Figure 5

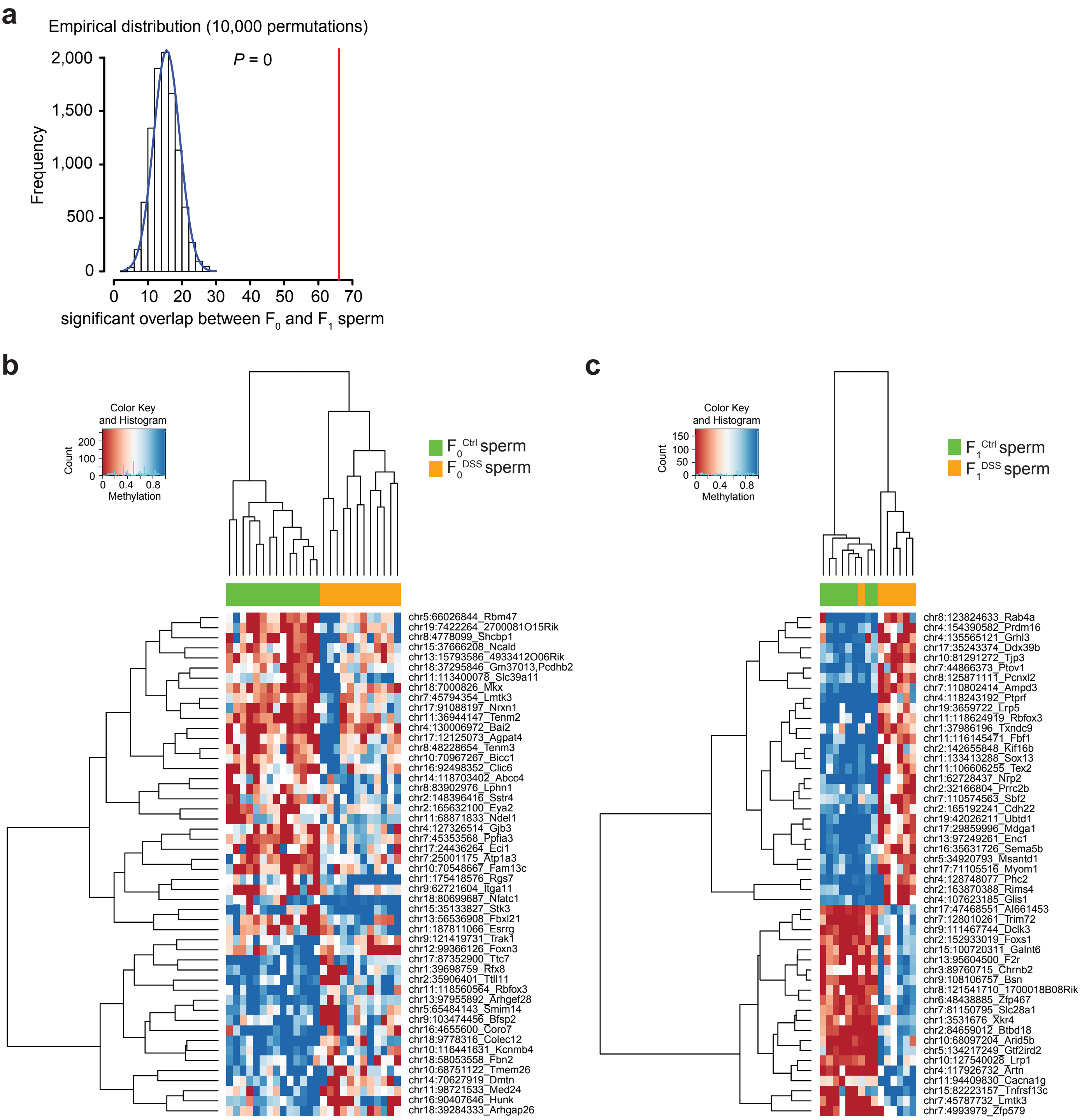

Supplementary Figure 6

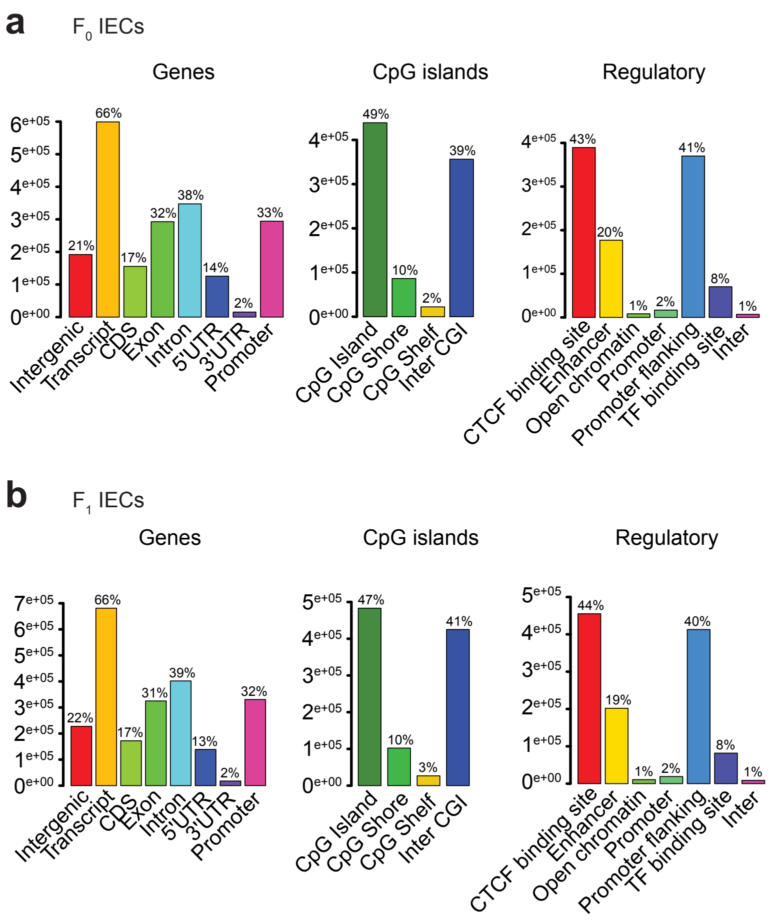

Supplementary Figure 7

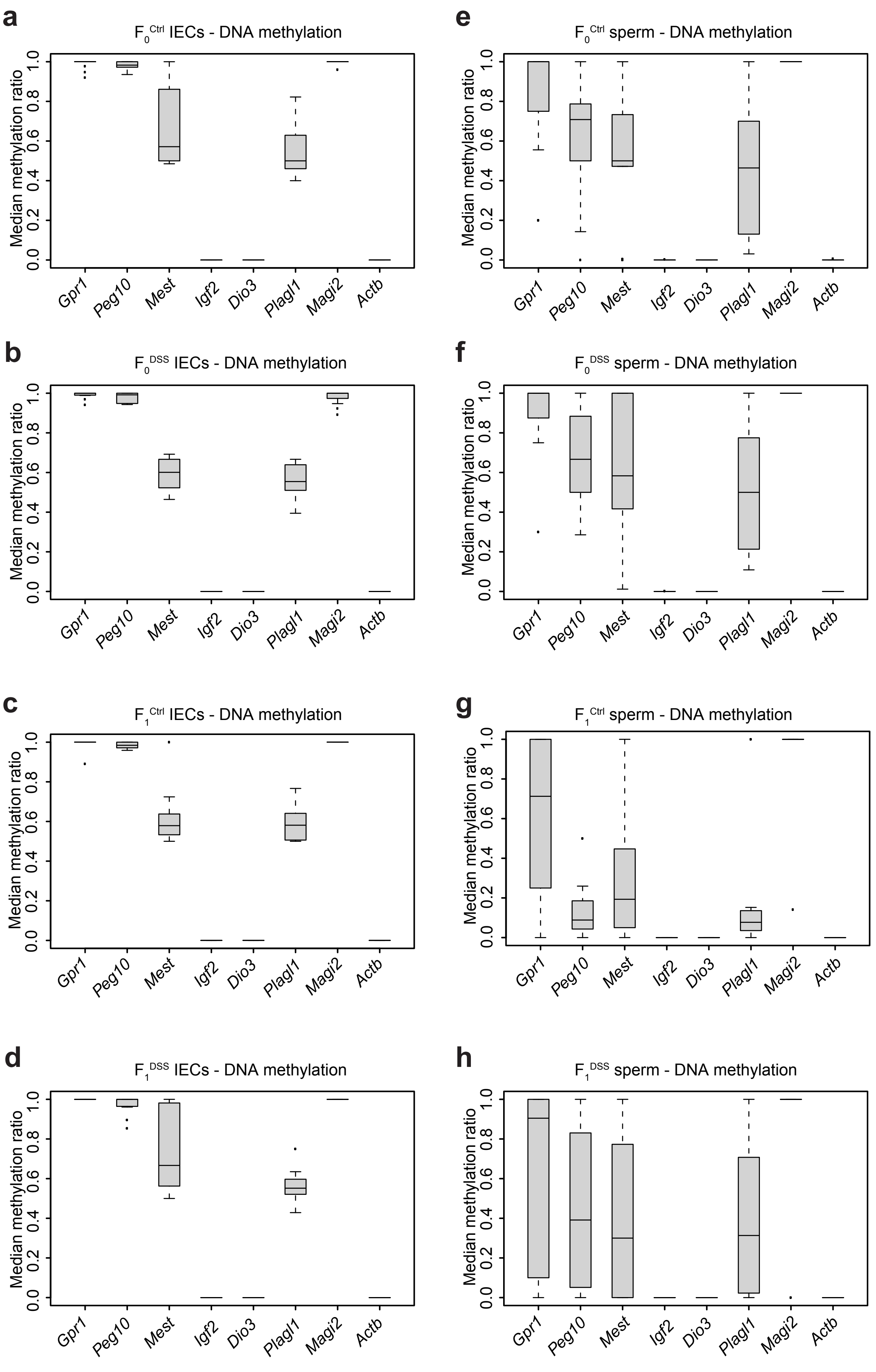

Supplementary Figure 8

a

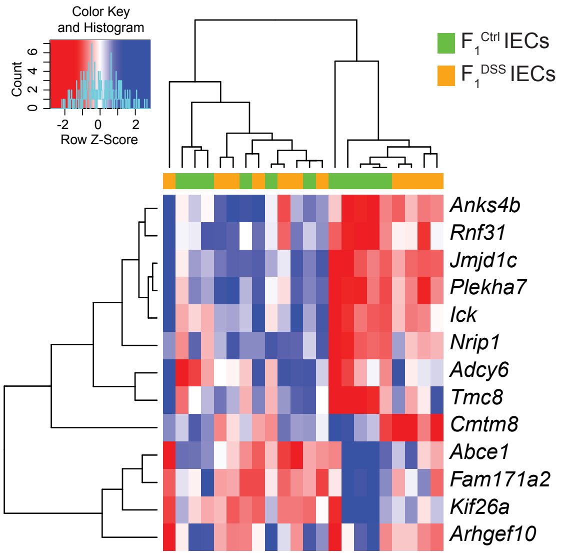

b

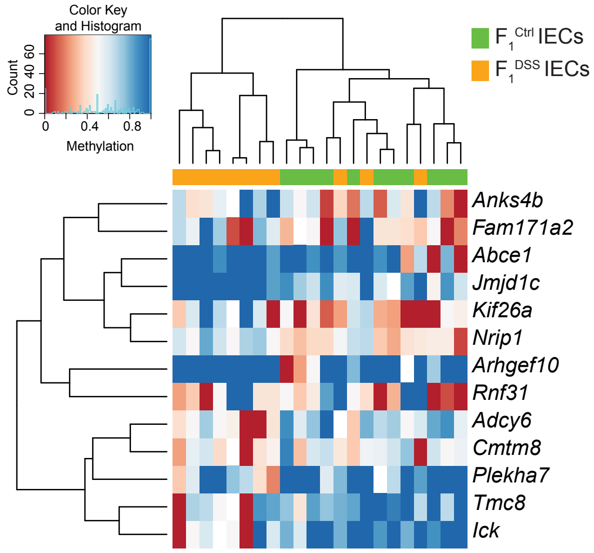

Supplementary Figure 9

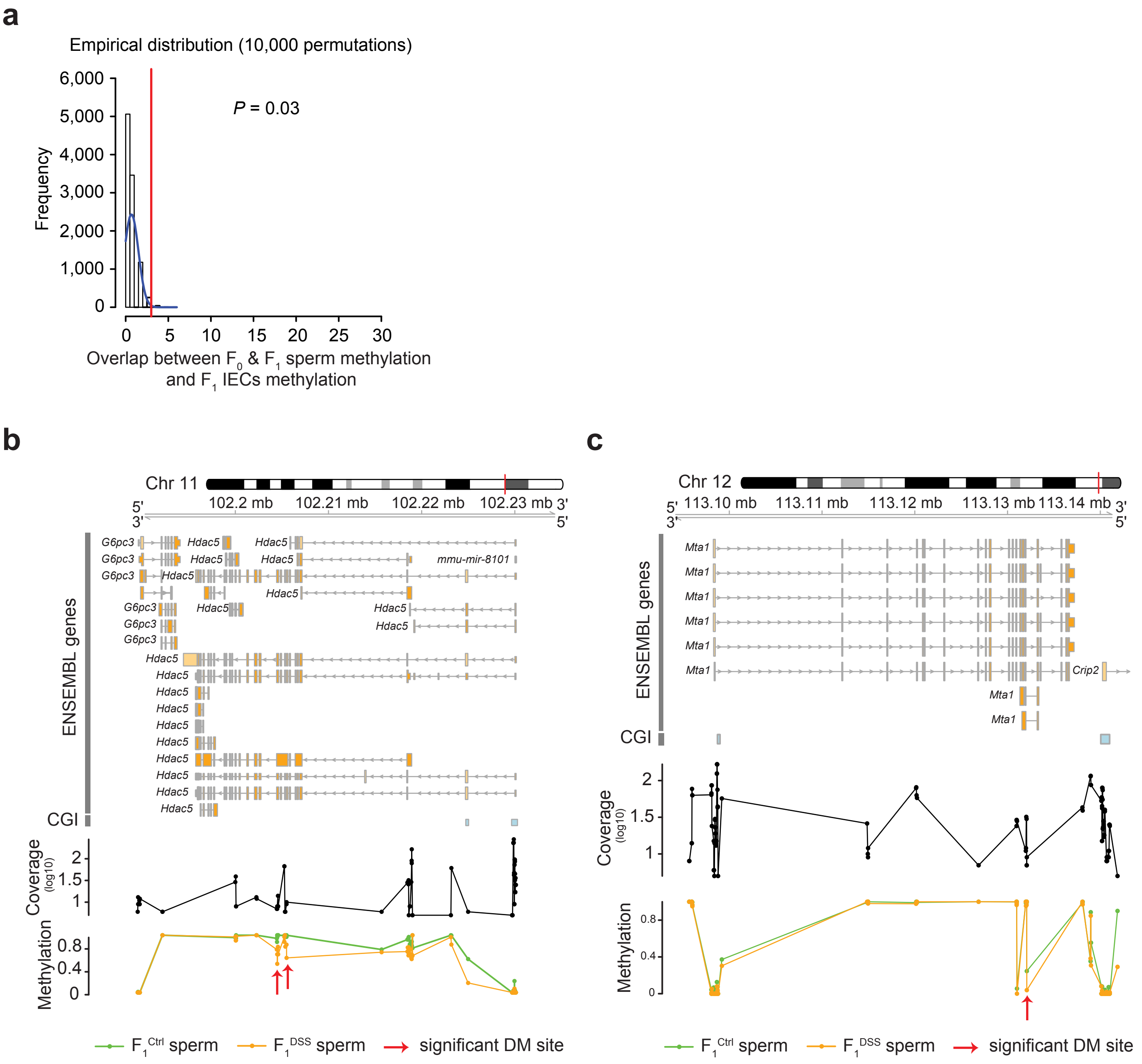

Supplement: Supplementary Information [file srep31640-s1.pdf]
